# If MIAVR is so good, why aren't we all doing it? A UK perspective

**DOI:** 10.1186/1749-8090-8-S1-O246

**Published:** 2013-09-11

**Authors:** Justin Smyth

**Affiliations:** 1 Dept. Cardiothoracic surgery, Royal Victoria Hospital, Belfast, UK

## Background

Minimally invasive aortic valve replacement (MIAVR) was first described in 1996 as an alternative to median sternotomy for AVR. Its uptake has been sporadic in cardiac surgery units in the UK. It remains unclear and somewhat controversial whether it has superior patient outcomes over conventional sternotomy. This survey aims to explore the reason for the low uptake by consultant surgeons in the UK and assess current opinions regarding the benefits, evidence base and barriers to MIAVR.

## Methods

An online survey was created with 20 questions designed to explore the reasons that the current consultant population uses to base its practise on MIAVR. The link was distributed by the UK Society of Cardiothoracic Surgeons to the consultant members. Opinions on benefits of MIAVR compared with conventional sternotomy were evaluated along with potential barriers and areas for future research. Information regarding use of MIAVR in their clinical practice was obtained.

## Results

Forty-nine consultants responded (response rate approximately 30%).67% of the consultants have performed MIAVR. 84% of the consultants identified that MIAVR was performed in their unit. 45% who identified themselves as performing MIAVR have carried out less than 15 procedures. 22% have carried out more than 26 procedures.

Only 48% of consultants felt there was an overall benefit of MIAVR when compared with conventional. Consultants with less than 10 years experience had a significantly more positive opinion of MIAVR than their more experienced colleagues.

## Conclusions

These results suggest that consultants’ opinions on the benefits and drawbacks of MIAVR remain divided. It has highlighted some perceived barriers and the desire for further evidence on which to base practice. Cost-benefit analysis would enable units to make an informed decision on the most effective approach to patients requiring AVR.

